# The current status of knowledge of herbal medicine and medicinal plants in Fiche, Ethiopia

**DOI:** 10.1186/1746-4269-10-38

**Published:** 2014-05-06

**Authors:** Elizabeth d’Avigdor, Hans Wohlmuth, Zemede Asfaw, Tesfaye Awas

**Affiliations:** 1 School of Health and Human Sciences, Southern Cross University, PO Box 157, Lismore, NSW 2480, Australia; 2 Division of Research, Southern Cross University, PO Box 157, Lismore, NSW 2480, Australia; 3 Department of Plant Biology & Biodiversity Management, College of Natural Sciences, Addis Ababa University, P.O. Box 3434, Addis Ababa, Ethiopia; 4 Ethiopian Institute of Biodiversity, PO Box 30726, Addis Ababa, Ethiopia

**Keywords:** Ethiopia, Herbal medicine, Traditional medicine, Ethnobotany

## Abstract

**Background:**

A majority of Ethiopians rely on traditional medicine as their primary form of health care, yet they are in danger of losing both their knowledge and the plants they have used as medicines for millennia. This study, conducted in the rural town of Fiche in Ethiopia, was undertaken with the support of Southern Cross University (SCU) Australia, Addis Ababa University (AAU) Ethiopia, and the Ethiopian Institute of Biodiversity (EIB), Ethiopia. The aim of this study, which included an ethnobotanical survey, was to explore the maintenance of tradition in the passing on of knowledge, the current level of knowledge about medicinal herbs and whether there is awareness and concern about the potential loss of both herbal knowledge and access to traditional medicinal plants.

**Methods:**

This study was conducted using an oral history framework with focus groups, unstructured and semi-structured interviews, field-walk/discussion sessions, and a market survey. Fifteen people were selected via purposeful and snowball sampling. Analysis was undertaken using a grounded theory methodology.

**Results:**

Fourteen lay community members and one professional herbalist provided information about 73 medicinal plants used locally. An ethnobotanical survey was performed and voucher specimens of 53 of the plants, representing 33 families, were collected and deposited at the EIB Herbarium. The community members are knowledgeable about recognition of medicinal plants and their usage to treat common ailments, and they continue to use herbs to treat sickness as they have in the past. A willingness to share knowledge was demonstrated by both the professional herbalist and lay informants. Participants are aware of the threat to the continued existence of the plants and the knowledge about their use, and showed willingness to take steps to address the situation.

**Conclusion:**

There is urgent need to document the valuable knowledge of medicinal herbs in Ethiopia. Ethnobotanical studies are imperative, and concomitant sustainable programmes that support the sustainability of herbal medicine traditions may be considered as a way to collect and disseminate information thereby supporting communities in their efforts to maintain their heritage. This study contributes to the documentation of the status of current traditional herbal knowledge in Ethiopia.

## Background

Ethiopia has been described as one of the most unusual and important sources of biodiversity in the world [1], yet is perilously close to losing much of this rich diversity due to deforestation, land degradation, lack of documentation of species in some areas as well as of traditional cultural knowledge, and potential acculturation [2-5]. Intertwined with the irretrievable loss of important species of animals and plants is the risk of loss of traditional herbal medicine knowledge.

An estimated 80 to 90 per cent of Ethiopians use herbal medicine as a primary form of health care [6-9]. Despite significant recent improvements in modern health care, many rural communities continue to have limited access to modern health care due to availability and affordability [10,11]. It is widely acknowledged that the wisdom of both professional and lay healers in applying traditional medicine to support health and manage illness may be lost to future generations unless urgent efforts are made to document and disseminate the knowledge [3,4,7,12,13] and to engage the younger generation who may no longer be interested in learning the traditional methods [4,7,14]. Therefore Ethiopians, particularly those in rural areas, face an uncertain future in regard to ready access to affordable modern medical services and access to their traditional remedies.

### Tradition

Herbalism is one aspect of traditional medicine practice in Ethiopia as it is in many other countries [15]. Herbs have traditionally been used in the home to treat family sickness, and occasionally traditional healers may be consulted. Traditional healers may be from the religious traditions of Cushitic Medicine, regional Arabic-Islamic medical system, or the Semitic Coptic medical system practiced by Orthodox Christian traditional healers [3], who are also referred to in Amharic as *debteras*. There may be many variations in approach within each system [16]. Spiritual methods are often used in combination with herbal applications particularly by the *debteras*, and the knowledge is traditionally passed down through the male line. When it comes to household herbal knowledge in the lay sphere, it is also generally considered that knowledge, in accordance with tradition, is preferentially passed on to a favourite child, usually a son [3,12,17,18], although a 2003 study by Gedif and Hahn [17] into the use of herbs for self-care, which primarily interviewed mothers, acknowledged mothers as the “de facto healers of the family treating accidents and ailments with medicinal plants”.

### Significance of the study

This study examined whether (i) knowledge was transferred to the current generation of lay community members in Fiche, (ii) lay people are knowledgeable about the medicinal use of herbs, (iii) lay people continue to practice herbal medicine in the treatment of sickness within the home. An aim of the study was also to determine whether or not there is enthusiasm for the preservation of knowledge and skills for future generations. The ethnobotanical survey that constituted part of this research helped to identify the plants used by local community members, for future planting in their household and community gardens. To our knowledge, no ethnobotanical exploration had previously been conducted in this area (personal communication, TA). The information gained from this study may inform further studies and projects aimed at documenting herbal knowledge in communities and supporting continued practice and sustainability of traditional herbal medicine in Ethiopia and elsewhere.

## Materials and methods

This case study was conducted using an oral history method, a technique for historical documentation which mirrors the cultural practice of passing on knowledge as an oral tradition, and encourages the subjects to present their experience of a specific event or period in the past [19]. It is a process of narrative building and within that framework the story of domestic life emerges. This gives contextual background to the information. A thematic analysis was applied to all interviews.

### Ethics

Official collaboration with, and permission from, the Ethiopian Institute of Biodiversity and Addis Ababa University to conduct research ensured that the collection of local medicinal knowledge was compliant with current Ethiopian regulations relating to Access and Benefit Sharing. Ethics approval (No. ECN-10-24) from the Human research Ethics Committee of Southern Cross University was granted, and verbal permission was sought from and granted by each informant, with full explanation given in the local language as to the purpose of the research. Permissions were recorded on film.

### Participants

**Figure 1:**
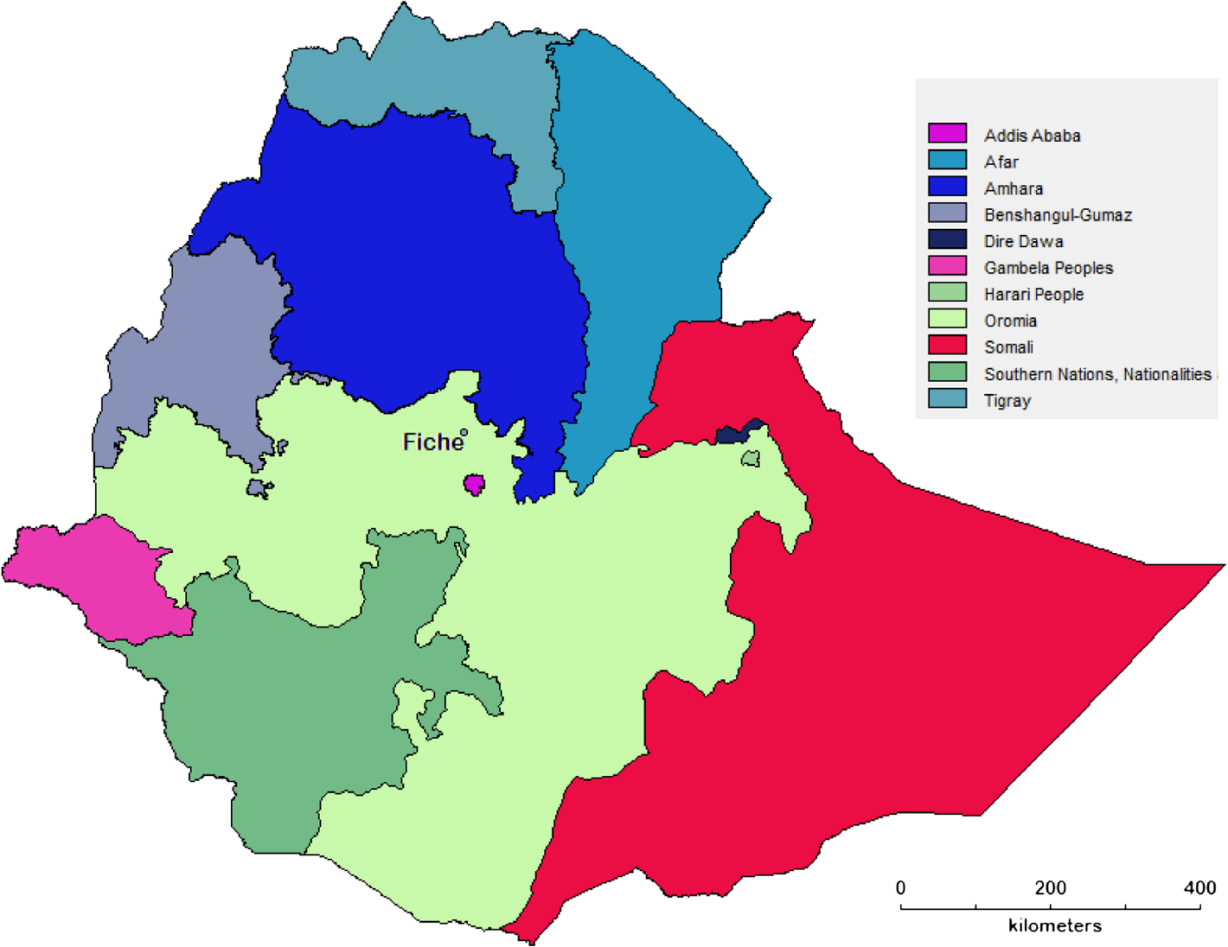
Map of Ethiopia showing Fiche.

The focus of the case study was the town of Fiche, in the North Shewa Zone of Oromia Region, Ethiopia. Fiche is located 115 km north of Addis Ababa, 9°48′N and 38°44′E, at an elevation of 2700 metres above sea level, with a town population in 2007 of 27,493 [20] (Figure 1).

Fieldwork was conducted in January and February 2011. Six informants were initially recruited via purposeful sampling by a tertiary-educated, local representative who is knowledgeable about local herbs (referred to herein as ‘M8’) and who is planning a herbal garden at Fiche (called “Doyu-Armon”). M8 speaks English and provided some translation. The criterion for the sampling was being known in the community to have knowledge of medicinal plants and their use to treat ailments. Further informants were recruited thereafter by snowball sampling. The 15 informants consisted of 14 community members (8 males and 6 females) and a professional herbalist (male) of the Ethiopian Orthodox Christian tradition. In addition to the professional herbalist, three of the males and two of the females were considered by the community to be particularly skilled in herbal knowledge. Informants were aged between 39 and 70, with an average age of mid-forties. Informants are referred to as Male (M) or Female (F) and assigned a number.

Informants’ education levels varied from illiterate (80% of informants), to secondary school education completed (10% of informants), with one tertiary-educated informant (M8, who initiated the recruitment of informants and provided some translation) and they belonged to either the Amhara or Oromo ethnic groups. All spoke Amharic and one (M8) was also fluent in English. In addition to the informants, some incidental data was contributed by one of the authors (TA of the Ethiopian Institute of Biodiversity) in his capacity as translator and collector of voucher specimens.

The first informants recruited (2 women and 4 men including the professional herbalist) were identified by the local representative (M8) as persons with significant relevant knowledge, and subsequent informants were recruited by snowball sampling. This sampling method was effective and convenient as it utilised local knowledge to identify appropriate informants.

The first focus group (FG1, six people) provided an introduction of the lead researcher to the community and established the reasons for her presence. Following this session, more people came forward, interested in being part of the process. The professional herbalist was considered a respected Elder and his encouragement to the group was evident. The field-walk/discussion sessions were conducted in two household gardens and the escarpment (open pasture) above the River Jemma Gorge. The market survey was conducted at the Saturday market in Fiche, and the information was obtained from the vendors of the herbs who were mainly women.

### Data collection

Field data were collected on six days during January and February 2011. A combination of focus groups (3), individual interviews (5), field-walk/discussion sessions (4) and one local market survey were conducted, with a tertiary-educated translator present at each session. Interview sites, all of which were in Fiche, were: Household garden (HG), homes of community members (H1 and H2), Doyu-Armon garden site (site for planned garden) (D-A), Escarpment above River Jemma Gorge (E) and Fiche Saturday market (M). The Jemma River is a tributary of the Blue Nile. Table 1 shows the timetable of fieldwork.

**Table 1:** Timetable of fieldwork

**Present:**	**Session**	**Where**	**Duration**
F1, M1, M8, R	Field-walk 1 (W1)	HG	1 hour
F1, M1, M3, M4, PH, M8, R	Focus group 1 (FG1)	H2	2 hours
PH, M8, R	Individual interview 1 (I1)	H2	1 hour
F1, E, M8, R	Field-walk 2 (W2)	HG	1 hour
	Collection of voucher specimens		
F1, E, M8, R	Field-walk 3 (W3)	HG	1 hour
	Collection of voucher specimens		
F6, E, M8, R	Individual interview 2/field-walk (I2)	Next to D-A on pasture	½ hour
	+ voucher specimen collection from Doyu-Armon garden site		
Female stallholders, E, R	Market survey (M)	M	1 hour
F4, F5, M8, R	Individual interviews 3 + 4 (I3)	H2	1 hour
M1, M2, M3, M5, M7, M8, F1, F4, F5, E, R	Focus group 2 (FG2)	H2	3 hours
M6, M8	Individual interview 5 (I5)	H1	20 minutes
M1, M3, M5, M7, M8, E, R	Field-walk 4 (W4)	E	2 hours
	Collection of voucher specimens		
F1, F2, F3, M1, M3, M5, M7, M8, E, R	Focus group 3 (FG3)	H	2 hours

Codes: F = Female, M = Male, PH = Professional Herbalist, E = Ethnobotanist (TA), R = Researcher (Ed’A), HG = Household garden, H1 and H2 = homes of householders, E = Escarpment above River Jemma.

Additional file 1 shows a plant collection site on the escarpment above River Jemma, as well as extracts of interviews.

The plant specimens collected by the Ethnobotanist (author TA) with the assistance of the informants were pressed, dried and identified following standard procedure and lodged at the EIB Herbarium in Addis Ababa. Translation was provided by TA and M8. All interview and focus group session translations were transcribed directly onto computer by the lead researcher, and all sessions were filmed, with the permission of participants. Later viewing of film footage provided useful review of data. In this way visual dynamics between informants could be viewed and further nuance from discussion picked up without the distraction of the recording process. Footage of 2 focus groups was viewed by a second translator to check areas where translation was indistinct, ambivalent, or not understood by the principal researcher. Other discussions, researcher observations and comments were recorded by hand into a notebook at the time, and a daily journal of all activities, with observations, comments and reflections, was written at the end of each day.

Interviews and focus groups were semi-structured. In an effort to ensure the women and men contributed equally during the mixed focus group discussions, an opening question (“How did you learn?”) was directed to each person individually. In this way, informants were able to provide in-depth answers in an individual manner as well as collectively. Occasional prompting, especially on the field-walk activities, would include the questions “What do you use this herb for?” How do you use this herb?” and “What do you call this herb?” allowing uninterrupted flow of discussion unless it strayed significantly from the topic, in which case an appropriate question was asked. Some contextual information was given by the free discussion in this way, often providing additional (unprompted) cultural background.

### Data analysis

Grounded theory was applied as a method to conceptualise the data and identify themes. Grounded theory is a method which allows themes to emerge through analysis of data and may provide further deep, thick context to a theory by exposing underlying processes [21]. In keeping with this approach to interpretive analysis, transcripts from each interview were analysed repeatedly to identify emerging themes, and concept codes were assigned (open coding). Coding formed the basis for categories, and the data were examined within categories. Seven category headings were identified and under these all the data were accounted for. Data were examined for herb names, for disease names, and for formulas or prescriptions, and a quantitative list constructed The existing literature was examined for documented uses in Ethiopia of the herbs mentioned and included in this list as a commentary.

## Results and discussion

Given that the research was conducted in a language and culture different from that of the principal researcher, some discussion of method with this aspect in mind is pertinent.

The intensive biography interview style of data collection associated with the oral history method allows a researcher to learn about informants’ lives from their own perspective [22]. The open discussion of memories, within the context of talking about herbs given to an informant as a child, gave the researcher the opportunity to observe and learn about informants within the context of their home life. Traditional medicine studies undertaken in Ethiopia are not often conducted in this way, with the perspective of an outsider exploring the current situation of the threat of loss of an important tradition, keeping cultural context at the forefront. Whilst being an outsider may on the one hand be seen as a limitation, on the other hand the researcher’s presence and interest in their plight highlighted outside interest and gave the community a sense that others considered their knowledge important and of value. The potentially negative issue of being an ‘outsider’ was ameliorated by the facts that the principal researcher is a herbalist in her own country, is able to speak a little of the language, was introduced to the community by a trusted member of that community and had previously visited Ethiopia (although not this area) on several occasions. The initiation of a programme to support establishment of a medicinal herb garden in the area (Botanica Ethiopia, see Additional file 2), also demonstrated tangible ongoing support to the community beyond the research programme.

According to Bryman [19], oral history testimonies have provided a method for the voices of the marginalised to be heard. It is not just people who may be marginalised, but also cultural traditions. In respect to the community group in Fiche, important cultural traditions and associated knowledge may be marginalised because community members may not have a strong voice in determining the future of those traditions. Further, the female members of this community may find their knowledge marginalised because despite the acknowledgement that women practice herbal medicine in the home [17,23], the prevalent belief [3,12,17,18] is that men (both professional traditional healers and in the family) are the prime holders of the knowledge. Time constraints of daily household chores may further restrict women’s participation in both receiving and passing on knowledge, and having that knowledge may not receive the importance it deserves [9].

The grounded theory approach to analysis was helpful, especially given the particular complexities associated with this study *viz.* the principal researcher was collecting data while immersed in a language, culture and environment different from her own. Repetition of certain words (translated) provided an opportunity to identify themes. For instance, the word “learnt” appeared at least once per person interviewed in describing different events, not surprising given the question asked but this provided a focus for analysis on first pass. In association with the words “learnt” or “remembered” would be a reference to a family member or influential person. The word “childhood” appeared frequently in this context. Another theme that emerged related to accessibility, availability and sustainability of herbs with subcodes referring to “disappeared”, “inaccessible”, “not available”, “hard to find”. Once emergent themes were identified, data were fragmented to lift coded elements out of the context of each interview [24] to list comments and information by group. Fragmented data were then reconnected and reviewed within the context of each interview. Throughout data collection, the researcher was critically aware that words emerged via translation and might have been influenced by translator bias. Mindful of this, the researcher would at times repeat the answer and ask for it to be translated back to the informants for verification. Table 2 lists the themes that emerged from coding.

**Table 2:** Themes Subthemes that emerged via the coding process were clustered into major themes

**Theme**	**Subtheme**
How knowledge is acquired from previous generation	People learnt from parents or other elders in the oral tradition
	People learnt from the treatment of their own illnesses as children
Awareness of loss of herbs	Now some herbs are difficult to access
	Some herbs are disappearing
	There is degradation of land
	Need to make effort to grow the herbs in household gardens
Conservation of herbs	Herbs need to be taken care of in the wild
	Wildcrafting is endangering some species
Passing on knowledge	Children may not be interested in learning about the herbs
	It is important to share the knowledge to save the herbs
Safety and dosage	Some herbs are toxic
	Some herbs are dangerous if combined
	Some herbs are dangerous if the dosage is too high
	Dosages adjusted for children
Gender	Women in general know more about application than men
	It is mostly women who sell the herbs in the marketplace
	Women have less time
Herb usage	Herbs are used in the home to treat family members for a range of illnesses or conditions
	Herbs are important
	Herbs are easily identified
	Herbs are sold in the market place

Fourteen lay community members (6 females and 8 males) and one professional herbalist provided information about 73 medicinal plants from 42 families. Voucher specimens of 53 of these, representing 33 families, were collected and deposited at the Herbarium of the EIB in Addis Ababa. The families contributing the most taxa were Asteraceae (6), Solanaceae (6), Lamiaceae (5) and Fabaceae (5). The major classes of indications cited by informants were gastrointestinal complaints (25 plants) including *megagna* (12), tapeworm infection (8) and hepatitis (5); psychiatric conditions (7) and respiratory complaints (5).

All herbs named, their uses, and a comparison with uses elsewhere in the literature, are shown in Table 3.

**Table 3:** Herb data chart

**Botanical and family name **[25]	**Local name (Amharic)**	**Voucher no.**	**Use**	**Preparation**	**Informant (code)**	**Quotes and observations**	**Literature**
*Achyranthes aspera* L. Amaranthaceae	*Telenj/qay telenj*	1933	Part of a recipe for *shotelay* (Rhesus factor incompatibility in pregnancy) combined with *Serabizu* (*Thalictrum rhynchocarpum*), *Quechine (Indigofera zavattarii*), *Y’imdur embway* (*Cucumis ficifolius*), *Tefrindo* (*Gomphocarpus purpurascens*), *Tult (Rumex nepalensis)*	The herbs are dried, chopped together and put in a cotton pouch to be hung around the pregnant woman’s neck in the seventh month. When the baby is born it is taken off the mother and put on the baby	M3	“To be collected on a Wednesday or a Friday, having abstained from sexual relations, and having not spoken to anybody on the morning of the collecting day. The herbs are dried outside the house, chopped together and put in a cotton pouch. The cotton must be spun by a lady in menopause, and spun with her left hand not her right hand. The pouch is put on the lady’s neck and as soon as she gives birth it is taken from her and put on the baby’s neck….this is my specialty“	Anti-fertility [26]
							Fresh pulverised leaf or its juice is placed in the nostril or its juice is sniffed for epistaxis. The crushed fresh leaf is also placed in the genitalia as a remedy for menorrhagia and to stop post-partum haemorrhage [27]
							Herpes zoster, blood clotting[28]
							Wound [29]
							Wound [30]
							Vaginal fumigation [31]
			Wounds (*kusil*)	Leaves rubbed and put on cut or wound	F1		
*Acokanthera schimperi* (A.DC.) Schweinf.	*Mrenz*	2016	Psychiatric disease (*likuft*)	Used in a formula (see *Solanum incanum*)	F3		Antiarrhythmic, vasoconstrictor, hypertensive agent, Na/K ATPase inhibitor [32]
Apocynaceae							
*Actiniopteris semiflabellata* Pic.Serm	*Menna*		Burn (severe)	Powdered roasted plant applied topically	M2	“It was immediately cured by a *shamagalay* (old man) around the church. The doctor’s treatment had not worked. I asked the *shamagalay* why this worked better than the clinic treatment. He said it was to contain the wound so that it did not affect the bone”	
Pteridaceae							
*Albizia anthelmintica* Brongn.	*Musena*		Taeniasis	*Musena* and *Enkoko (Embelia schimperi)* given but not in combination	M7	Drink either with *tella* (local beer)	Bark powder is cooked with meat and soup is taken as tenifuge [33]
Fabaceae				The bark is mixed with *Nug* (*Guizotia abyssinica*) and sugar	M5		
				The bark is mixed with *Nug* (*Guizotia abyssinica*), chopped together	F2	“If you take *musena* you may never see the segments…it kills all internally, it is digested. There will not be another infection”	
					F1, F4	“We buy the *Musena* from the market”	
*Allium cepa* L.	*Shinkurt*		Taeniasis	As part of a formula comprising Arake (spirit brewed with fermented grains) with *Kosso (Hagenia abyssinica), Tenadam (Ruta chalepensis), Zingibil (Zingiber officinale)* and *Quorofa (Cinnamomum verum)*	F2	The herbs are used in the brewing of Arake	Widely used as a medicinal plant worldwide
Amaryllidaceae							
*Allium sativum* L.	*Nech shinkurt*		Asthma	3-4 cloves chopped and mixed with honey, dissolved by *Kosso arake* (spirit brewed with fermented grains and *Hagenia abyssinica*)	F2	“The *Kosso arake* dissolves the *Nech shinkurt.* The *Nech shinkurt* can have a kind of side effect on the stomach (gastritis). If you want to protect yourself you may take lightly roasted *Talba* (*Linum usitatissimum*) or *Abish (Trigonella foenum-graecum)*”	For common cold, malaria, cough, lung TB…asthma…parasitic infections, diarrhoea (etc.) [34]
Amaryllidaceae							
							Widely used as a medicinal plant worldwide
*Aloe debrana*			Wounds (*kusil)*		M1	“A wound that is infected and very dry, contracted, they will use *Aloe debrana* and it will relax”	
Christian							
Xanthorrhoeaceae							
*Aloe pulcherrima* M.G.Gilbert & Sebsebe	*Sete eret*	2000	Asthma	The sap is boiled with water. Sugar is added. This is filtered to about ½ teacup. Drink this and suck on a lemon. Do this for four days.	M6	“You will burp the lemon taste, not the bitter aloe taste. After using this recipe I am free from asthma”	“The species grows….in Gonder, Gojam, Welo and Shewa floristic regions. It is so far not known anywhere else. It occurs in a very sporadic manner, mainly on cliffs, and almost always in inaccessible places” [35]
Xanthorrhoeaceae							
*Aloe* spp.	*Eret*		Burn	The burn is washed first with warm water and salt, then *Eret* placed on top	F1	“We do not use alcohol to wash it like the doctors do”	
Xanthorrhoeaceae							
*Andrachne aspera* Spreng*.*	*Tekeze*		Unexplained stomach ache *(megagna*)	The root is chewed for stomach treatment and nausea (anti-emetic)	M1, F1	“Not during pregnancy”	Ascariasis, stomach distention, malaria, asthma, gastritis, liver disease and as anti-emetic [36]
Phyllanthaceae			Snake bite	The root is chewed, followed by lots of water. Will cause to vomit	F1		
*Artemisia absinthium* L.	*Ariti*	2024	Unexplained stomach ache (*megagna)*	Mixed with *Tej sar* (*Cymbopogon citratus*) and made into an infusion and filtered, and drunk	F4	“*Ariti* tastes bitter, like *Kosso*” (*Hagenia abyssinica*)	The juice of the powdered leaves is taken with honey to treat stomach ache [37]
Asteraceae							
				Mixed with *Tenadam* (*Ruta chalepensis*), and *Zingibil* (*Zingiber officinale*) made into an infusion, filtered and drunk	M3	Remembers *megagna* as a childhood illness. “The pain immediately disappeared when this mixture was drunk”	Cholagogic, digestive, appetite-stimulating, wound-healing, anticancer, antiparasitic [38]
						Found on sale in Fiche market, as part of a fragrant bouquet (with *Tej sar – Cymbopogon citratus, Ujuban – Ocimum basilicum* var. *thyrsiflorum,* and *Tenadam – Ruta chalepensis)*	
*Artemisia abyssinica* Sch.Bip. ex A. Rich Asteraceae	*Chikugn*	1999	Evil Eye, combined with *Tenadam* (*Ruta chalepensis*) and *Shinkurt* (*Allium cepa*)	Take the dried skin of a hyena and put the herbs in a pouch of the leather as a charm around the neck.	M6	“I used to suffer from evil eye in childhood. If that is prepared and is smelling in the house, someone who is suffering from evil eye will start shouting and moving around; they will tie him down by force and apply in his nose. If you apply this, he will tell you the person with the evil eye up to the seventh generation”	Anti-leishmanial, intestinal problems, bronchitis and other inflammatory disorders, cold and fever, anorexia, colic, infectious diseases (bacterial, protozoal), headache, amenorrhoea and dysmenorrhoea [39]
							Eye infection – topically [40]
			Psychiatric disease (*lekeft*)		F1		Haemostatic (nose), tonsillitis, cold, constipation, rheumatism [41]
				Take *Chikugn* (*Artemisia abyssinica*) *and three young leaves of Set eret (Aloe pulcherrima)* with *Nech shinkurt* (*Allium sativum*), *Tenadam* (*Ruta chalepensis*), the whole plant of *Tekeze* (*Andrachne aspera*), along with the leaves of *Chat* (*Catha edulis*) and *Ye* a*hiya joro* (*Verbascum sinaiticum):* chop together. The juice is applied to the nose	F2	“My father was told by somebody”	
							Whole herb is use for tonsillitis [42]
			Fumigant for milk machinery		F1		
*Asparagus africanus* Lam.	*Seriti*	1928	Rituals such as circumcision, and giving birth	Branch hung in the doorway	M1	Considered cleansing because “women are unclean just after giving birth”	Fresh pulverised root taken mixed with water to stimulate milk secretion. The use of the plant against gouty arthritis and as abortifacient have been recorded [43]
					M8		
Asparagaceae			Hung on the door where *Tella* (local beer) is being made, as protectant against uncleanliness (someone who is menstruating, or has recently had sexual relations)		F1		
*Brucea antidysenterica* J.F. Mill	*Fit aballo, aballo*	2013	Eczema (*chiffe*)	The leaves are collected and dried, the powder is then applied to the skin	F2	“I had this disease in childhood”	*Bullad* (weight loss, fever, itching, diarrhoea) [28]
							Evil eye (tied around neck) [30]
Simaroubaceae							
							Cancer treatment, diarrhoea, evil eye, leishmaniasis, rabies, scabies, skin disease, wound [12]
*Calpurnia aurea*(Aiton) Benth.	*Digita*	2008	Child with diarrhoea (*tekmet*)	The leaves of the young shoots from seven plants of *Digita* are rubbed in the hands for the juice; the juice is mixed with water Dosage is very important, depending on the age of the child	F3	“5 year old, 1 teaspoon, just once. This is what I had as a child”. Some discussion about the toxicity of this plant	Decoction of the fresh leaf has been used against hypertension. Quinolizidine alkaloid, calpurnine, has been isolated [44]
Fabaceae							
					M1	“The stem bark is poisonous. The dosage should be measured carefully. Only the young shoots are used. Even then one has to be very careful.”	
							Diarrhoea [45]
					F1	“You can become crazy from it. If you go crazy, then you are going to die”	Amoebiasis, giardiasis [30]
							*Kuruba* (diarrhoea) [28]
							Used as a fish poison or as a cure for dysentery [46]
*Capparis tomentosa*	*Gumero*		Psychiatric disease	In formula (see *Solanum incanum*)	F3		Bleeding after delivery [30]
Lam.							
Capparaceae							
*Catha edulis* (Vahl) Endl.	*Chat*		Psychiatric disease	In formula (see *Artemisia abyssinica*)	F2	Frequently observed sold in streets	Ephedrine has been isolated from this plant. Possesses psychostimulant properties [47]
Celastraceae							
*Chenopodium murale* L.	*Sinko*	1930	Unexplained stomach ache (*megagna)*	The young shoots are collected with scissors and rubbed through a sieve as used for the domesticated grass *Tef* (*Eragrostis tef*)	F4		
Amaranthaceae							
*Cinnamomum verum* J. Presl	*Qorofa*		Taeniasis	In formula (see *Allium cepa*)	F1		
Lauraceae							
*Croton macrostachyus* Hochst. Ex.Delile Euphorbiaceae	*Bisana*	2007	Skin rash	Mixed with egg yolk and applied to the skin	F1		Aphasia, ascariasis, constipation, eye disease, haemorrhoid, induction of abortion, purgative, ringworm, taeniasis, stomach ache, venereal disease control [12]
			Skin rash	The fresh bud is cut and the fluid applied to the rash. If the problem is on the head, the head is shaved and bud fluid applied	M8		
			Dandruff				Scabies, *kuruba* (diarrhoea), hepatitis, Tinea versicolour [28]
							Malaria [30]
*Cymbopogon citratus* (DC.) Stapf	*Tej sar*	2004	Unexplained stomach ache (*megagna)*	Mixed with *Ariti* (*Artemisia absinthium*)	F5	Found at the marketplace as part of a fragrant bouquet	Treatment of heart, chest and stomach complaints [48]
Poaceae							
							Stomach ache, smallpox, common cold [12]
							Ascariasis [30]
*Datura stramonium* L.	*Astenagir/Astenagirt*	1940	Hallucinogenic		M8, E		Eye disease (‘crying eyes’) (topical), bad breath (smoke inhaled, fungus infection of the head (topical), mumps (topical), relief of toothache (vapour inhaled), rheumatic pain (vapour inhaled), treatment of burn (topical), wound (topical) [12]
Solanaceae							
							Swelling (topical), toothache (inhalation), dandruff (topical) [49]
							Swelling, toothache, dandruff, wounds [28]
*Echinops kebericho* Mesfin	*Kerbericho*	2001		To dispel nightmares in children	E	Found on sale in Fiche marketplace	Constipation, headache, heart pain, stomach ache, typhus [12]
Asteraceae							
							Fumigant after childbirth. Typhus fever. Stomach ache. Snake repellent in the house. Intestinal pains [50]
							Lung TB, leprosy, syphilis [51]
							Cough [49]
							Evil eye [28]
*Embelia schimperi* Vatke Myrsinaceae	*Enkoko*	2032	Taeniasis	Chopped with *Musena* (*Albizia anthelmintica*) and *Nug* (*Guizotia abyssinica*) and eaten	M3		Powder of fruit mixed with water and taken as taenicide [52]
				With *Musena* (*Albizia anthelmintica*) and *Nug* (*Guizotia abyssinica*), taken with a drink of *Tella* (local beer)	M1	“Must be taken simultaneously with *Tella*. Drink, then jump up and down to dissolve internally. (M7)	Taeniasis, disinfectant [12]
							Taeniasis, ascariasis [48]
							Tapeworm [30]
					M5	If not taken with *Tella*, you will become dizzy and fall” (M5)	
					M7		
					M2		
					M1	*“Enkoko* and *Musena* are both deadly”	
				With *Meterre* (*Glinus lotoides*) and *Kosso* (*Hagenia abyssinica*)	M8	“I remember my mother giving me this combination”	
				The ripe fruits are collected and the exocarp removed. Fruit swallowed directly using water	F2		
					M3	“It is ok to take *Enkoko*, *Musena* and *Nug* together”	
*Eucalyptus globulus* Labill.	*Nech bahirzaf*	2027	Fever with headache (*mich*), colds	Apply rubbed leaves directly to nose	F5		Leaves are boiled with water and the vapour inhaled to treat cough, flu and sore throat [53]
Myrtaceae							
*Euclea racemosa* L.	*Dedaho*	2028	Warts of the rectum	The root is to be collected early in the morning before urination. The root is dug up then boiled, and a full small teacup of the filtrate must be drunk before food. After the medicine is drunk well prepared food is eaten and well prepared *Tella* (local beer) is drunk	M6	“Finally a kind of faeces will come out. If this does not happen initially, then the process is repeated the next day”	Gonorrhoea, uterine prolapse, haemostatic, gastritis, diarrhoea, cataract, acne, chloasma, eczema, constipation, rabies, vitiligo, epilepsy [54]
Ebenaceae							
*Euphorbia tirucalli* L.	*Qencheb*	2026	Scorpion bite	The skin around the bite is slashed, and the milky sap applied	M5	“The scorpion has a venom that gives gland pain for three days. After this application I was ok. Previously with a bite I suffered for three days. This time I was back at work in three hours. I had a small glandular response this time”	Reported use in India for scorpion bite [55]
Euphorbiaceae							
*Galium simense* Fresen.	*Chogogit*	1998	Skin fungus (*qworqwor)*	The leaf is rubbed to get the juice which is applied to the affected place; the plant is then discarded. When applied, it irritates and causes a little bleeding. The next day it is washed off, and the patient has to wear newly washed clothing	E	“It will never come again”	Extract of fresh leaves and inflorescences is used in Ethiopia to dress new wounds and cuts [56]
Rubiaceae					M1		Snake bite [13]
					M6		
					M8		
*Glinus lotoides* L.	*Meterre*	2031	Taeniasis	Mixed with *Nug* (*Guizotia abyssinica*) and *Musena* (*Albizia anthelmintica*). Taken orally as a paste	F1, F5		Ascariasis, taeniasis, diabetes [12]
Molluginaceae							
				Cleaned and ground with *Nug* (*Guizotia abyssinica*), added sugar and eaten before food. Fast until noon before taking it, then the first meal afterwards should be soup.	E	Found on sale in Fiche market	Tapeworm – fruit powder mixed with *Nug* is taken orally [28]
				*Meterre* with *Nug* OR *Musena* with *Nug*	M1, M7, M8	Remembers mother giving him all three	
				*Meterre, Enkoko (Embelia schimperi) and Nug (Guizotia abyssinica)*	M8		
*Gomphocarpus purpurascen*s A. Rich.	*Tefrindo*	2005	Rhesus Factor problem in pregnancy (*shotelay*), as part of formula		M3		
Asclepiadaceae			(see *Achyranthes aspera*)				
*Guizotia abyssinica* (L.f.) Cass.	*Nug*		Taeniasis	Used as a binder with many preparations, mentioned here for tapeworm infection	F1	Found on sale in Fiche market	
Asteraceae							
					M1		
					M2		
					M3		
					M5		
					M7		
					M8		
*Hagenia abyssinica* J.F. Gmel	*Kosso*	2025	Taeniasis	The flower taken with *Tenadam* (*Ruta chalepensis*), *Shunkurt* (*Allium cepa*), *Zingibil* (*Zingiber officinale*) and Q*orofa* (*Cinnamomum verum*)	F1		Female flowers are employed as a taenicide against *Taenia saginata*[57]
							Eye disease, hypertension, scabies, m[12]
Rosaceae							
							Provides a strong and widely used anthelmintic [46]
*Hordeum vulgare* L. Poaceae	*Gebs*		Hypertension	Taken as a fermented barley drink. *Gebs* (germinated barley), *Mashilla* (*Sorghum spp*.) are baked together like a bread. This is broken up and fermented together with *beqil* (malt starter), brewed and distilled. Drunk from a shot glass	F1		Hordenine with diuretic and in large doses with hypertensive action has been isolated [58]
*Indigofera zavattarii* Chiov*.*	*Quechine*		Rhesus factor problem in pregnancy *(shotelay)*	In formula: see *Achyranthes aspera*	M3		
Fabaceae							
*Jasminum grandiflorum* L.	*Tembelel*	1957	Abdominal pain	The root is chewed	M1		
Oleaceae							
*Laggera tomentosa* (Sch.Bip.)	*Shiro kese*	1943	Unexplained stomach problems (m*egagna*)	Leaves crushed and inhaled	PH		
Asteraceae							
*Laggera crispata* (Vahl) Hepper & J.R.I. Wood	*Ras kebdo*	1929	Dandruff (*forefore)*	Leaf rubbed and applied to the scalp	F5		
Asteraceae							
*Leonotis ocymifolia* (Burm.f.) Iwarsson Lamiaceae	*Feres zeng*	1942	Headache (*ras metat*)	The collected leaves are rubbed between hands and put into nostrils to inhale	F6	“Particularly for headaches with tonsillitis. It cures it well. If not, the patient should be taken to the doctor. Go to a traditional medicine healer for headaches with tonsillitis”	
				OR			
				The juice is squeezed out and drunk with coffee.			
			Ulcer of the neck (*nkersa*)	Chopped leaves are applied to the ulcer for 24 hours	M7	“People here assume it is cancer of the neck, but it is an ulcer. My uncle tried many things but finally he cured me with this”	
			For sick chickens	With *Aya joro* (*Verbascum sinaiticum*)	F1		
*Lepidium sativum* L. Brassicaceae	*Feto*	2020	Unexplained stomach problems (*megagna)*	Ground, mixed with lemon juice and water	F5	Found on sale in Fiche market	Skin problems, fever, eye diseases, amoebic dysentery, abortion and asthma, intestinal complaints [59]
							Aphrodisiac, gastritis, headache, ringworm, *buda beshita* (evil eye) *mich* (fever with headache) [12]
							Stomach ache [30]
*Leucas abyssinica* (Benth.) Briq.	*Aychedamo*	1941	Eye infection		E		For eye diseases, twigs of Leucas abyssinica are crushed and coated on eyes [60]
Lamiaceae							
*Linum usitatissimum* L*.* Linaceae	*Talba*		Demulcent	Option as protective against gastritis when used with *Allium sativum* in treatment for asthma	F2	Found on sale in Fiche market	
*Lippia adoensis* Hochst. Ex Walp. Var.*Koseret* Sebesebe	*Koseret*	1931	Bee attractant		F1	Found on sale in Fiche market	Dried leaves powdered together with barley eaten to get relief from stomach complaints [61]
Verbenaceae							
							Malaria, fever, aphrodisiac [62]
*Malva verticillata* L. Malvaceae	*Lut*	1935	Expulsion of placenta in cow	The root is dug up and chopped and given as a decoction to cow	F6		
*Maytenus arbutifolia* (Hochst. Ex A. Rich.) R. Wilczek	*Atat*	2023	Psychiatric disease (in formula – see *Solanum incanum*)		F3		A number of *Maytenus* spp. Are used in traditional medicine to treat various disorders including tumors. A tumor inhibitor, maytansine, has been extracted [46]
Celastraceae							
*Myrsine africana* L. Primulaceae	*Kechemo*	2022	Taeniasis	Fruits are collected, chopped and filtered. Filtrate is drunk to expel tapeworm	F2	“If *Kechemo* does not work, go for one of the other ones – *Musena (Albizia anthelmintica*), *Enkoko* (*Embelia schimperi*), *Kosso* (*Hagenia abyssinica*)”	Fruit powder paste with Nug seed is taken against tapeworm and ascariasis [63]
							Twigs used as a toothbrush [46]
*Nicotiana tabacum* L. Solanaceae	*Tembaho*	2029	Repels snakes from garden		F1, E		
*Ocimum lamiifolium* Hochst. Ex Benth. Lamiaceae	*Demakese*	1926	Fever with headache (*mich)*	Rub in the hand and squeeze to get juice, add to coffee or drink	F5	Demonstrated putting a *gabi* – heavy cotton shawl – over the head for inhalation of vapour	The fresh leaves are squeezed and the juice sniffed to treat coughs and colds. The juice is also used as eye rinse to treat eye infections. The crushed leaves are put in the nostrils to stop nose bleeding [64]
			Influenza or cold	OR			
			Fever with headache	Boil the leaves, place on a hot iron pan and inhale the vapour		Found on sale in Fiche market	Cough, cold, headache, eye infection, hematuria, *mich* (fever with headache) [12]
				OR			
				Apply rubbed leaves directly into the nose			
				Juice in coffee	F6	“If the juice of *Demakese* is red when the herb is rubbed by a person, then the person has *mich*. If it is green, it is not *mich*. The mother or the daughter will apply this”	*Kusil* (wound), *mich* (fever) [28]
							*Mich *[29]
							*Mich *[4]
*Opuntia ficus-indica* (L.) Mill. Cactaceae	*Culcal*		Haemorrhage in childbirth	In a formula (see *Periploca linearifolia*)	M3		
*Otostegia fruticosa*	*Tinjut*	1932	Unexplained stomach ache (*megagna)*		F1	Found on sale in Fiche market	Insecticide, disinfectant, as a fumigant [12]
(Forssk.) Schweinf. ex Penzig							
Lamiaceae							
*Periploca linearifolia* Quart.-Dill. & A.Rich. Apocynaceae	*Tikur hareg*		Haemorrhage in childbirth	Combined in a formula with *Culcwal* (*Opuntia ficus-indica)* and *Qeret* (unidentified). All are chopped together and then the juice is collected separately (filtered), used as ink to write on paper as a charm hung around the neck	M3	“The *debtera* will write a charm with the filtrate and put it on her neck, and the blood will stop”	
			Prepared by *debtera*:	A potion is prepared, buried in the ground for a week. When opened, the inky fluid is used as an ink to write a spell, or charm. Alternatively, the ink is used to tattoo into the skin with a needle	M3	“The *debtera* will use this with other herbs to make a potion. This is put in a bottle and buried for seven days before September 11 (*Addis amet –* New Year’s Day). When opened it will have an inky constituency. The *debtera* will then use a pen made from *Arundo* (bamboo), and will write on white paper. It is then worn on the neck. Another way is to tattoo the ink into the skin with a needle”	
			To keep the wife from straying				
			To stop enemies from attacking				
			To prevent bullets from penetrating				
			To keep devils away				
			To stop pain				
*Phytolacca dodecandra* L’Herit	*Endod*	1927	Bilharzia		E		Molluscide against Bilharzia [46]
			Contraception	The whole roots of 7 young plants without branch, flower or fruit (sterile) are collected, being careful to get it all, on a Friday or a Monday. These are chopped and then mixed with honey, which is collected in October. The woman should take it at the end of menstruation	F1	Debate on this application. Some say the woman should sleep with her husband on the day she takes the medication. “If she sleeps with her husband the ovary will not be badly affected” (M1). “If she goes to the doctor they will clean up that one and she will become pregnant” (F1). “She has to continue sexual relations to stop her ovary being badly affected” (M1). “She has to go to hospital” (M3). Some say it does not matter; used as a contraceptive, the woman will stay without child for 5–6 years. If she wants to become pregnant, she has to take an antidote (*merfchow)* – another plant. M7 says “If she takes the *endod* she is permanently sterile”. F1 says “If you spray poison on a flower, it will die”. M2 says “I gave it to my wife and 18 other people. No-one has given birth after that. My wife now wants to have a baby and cannot”	Ascariasis, eczema, gonorrhoea, infertility, liver disease, malaria, rabies, soap substitute, syphilis [12]
Phytolaccaceae					M1		
					M2		
					M3		Rabies [4]
					M7		
			Skin blisters (*ekek*) – viral infection	The chopped fruit is mixed with water as a wash for the hands	M8		
*Podocorpus falcatus* (Thunb.) R.Br.ex Mirb. Podocarpaceae	*Zegba*		Hepatitis formula	Formula:	M2	“My uncle took the leaf of *Zegba* and leaf of *Togor* and leaf of *Nechilo.* Then the root of *Chifrig* and the young shoot of *Yerzingero addis* and then *Embwacho* and the whole plant of *Serabizu* and the young shoot of *Gesho*. All this was put together, chopped, added to water and stirred. This is applied to whole body of the child every morning for seven day, starting on a Wednesday or a Friday and it must be a cloudy day. But it must not be too cloudy”	Four species of *Podocarpus* including *Podocarpus falcatus* all exhibited strong inhibition against *Bacillus subtilis, Staphylococcus aureus, Escherichia coli, klebsiella pneumonia* and *Candida albicans*[65]
				Zegba			
				Togor leaf (unidentified)			
				Nechilo (unidentified)			
				*Chifrig (Sida massoika)*			
				*Yezingero addis* (unidentified)			
				*Embwacho (Rumex nervosus)*			
				*Serabizu (Thalictrum rhynchocarpum)*			
				*Gesho (Rhamnus prinoides*)			
				Topical application			
*Polygala hottentotta*	*Etse adin*	1996	Anti-venom		F5		
C. Presl							
Polygalaceae							
*Rhamnus prinoides* L’Herit	*Gesho*	1952	Hepatitis (in formula)			Found on sale in Fiche marketplace	
Rhamnaceae							
*Rhus retinorrhoea* Steud. Ex A.Rich. Anacardiaceae	*Tilum*	2009	Wounds	Rubbed in hands and then put on wound	M4		
*Rumex abyssinicus* Jacq. Polygonaceae	*Mekmeko*	2012	Hypertension		F1		Gonorrhoea, lung TB, leprosy, fever [66]
							Itching skin [4]
							Extracts drunk to control ‘mild form of diabetes’ [46]
*Rumex nepalensis* Spreng*.* Polygonaceae	*Tult*	1936	Unexplained stomach ache (*megagna*)	The root is dug out and chewed. If *Tult* is not available, then the leaves of *Tenadam* (*Ruta chalepensis*) may be used instead	M2	Childhood memory of use. “*Tult* is very bitter. I was forced to chew it, I would be beaten if I did not chew it”	Amoebiasis, tonsillitis, uterine bleeding [12]
							Abdominal cramp, child diarrhoea, toothache, liver disease, eye infection [4]
							Stomach ache [13]
			Rhesus factor problem in pregnancy	Part of formula (see *Achyranthes aspera*)	M3		
*Rumex nervosus* Vahl.	*Embwacho*	2011	Eye problems	Leaves are collected, dried and pounded	F5	Remembers this from childhood	For dysentery, roots powder of *Rumex nervosus* mixed with melted butter. Stomach ache, roots in a honey paste dressing. Warts (*kintarot*), roots powder on cut edge [49]
Polygonaceae			Wound *(kusil)*				
			Hepatitis	In formula (see *Podocarpus falcatus)*	M2		
			Roundworm	Stem chopped with salt	M1	‘My father collected *embwacho* and he kept a stem and chopped it in small pieces, added salt, gave it to me and forbade me from eating for one hour. After three days there was expulsion of worms and no problem since then”	
*Ruta chalepensis* L.	*Tenadam*	1997	Unexplained stomach ache (*megagna*)	In formula (see *Artemisia absinthium*)	M3	“The pain immediately disappeared”	Snakebites, headaches, abdominal pain, strained eye, head lice, fever, poor blood circulation, local paralysis, nervous tension, cough, asthma, infected wound, rheumatism. An infusion is also used as a tea to treat headaches, cold, heart pain, earache and intestinal disorder. Dried fruits boiled with milk are used against diarrhoea, or with *Tella* (local beer) or “*wet*” (stew) against influenza [67]
Rutaceae				Chew the leaves	M2, M4	Use if *Tult* (*Rumex nepalensis*) not available	
				Combine with *Dingetegna (Taverniera abyssinica)* and wood ash mixed with a little water	F1	Will cause to vomit	
			Colic in baby		M3	*Tenadam* and *Ariti* (*Artemisia absinthium*) have the same use for treating the stomach”	
					PH	Found on sale in Fiche marketplace	Stomach problems [68]
							Evil eye and ‘flu’ [28]
*Sansevieria ehrenbergii* Schweinf. Ex Baker	*Wonde cheret*		Ear infections		F1		
Asparagaceae							
*Sida massaica* Vollesen	*Chifrig*	1956	Roundworm	The whole part is ground and made into an infusion, filtered and drunk	F5		
Malvacaeae			Hepatitis	In formula (see *Podocarpus falcatus)*	M2		
*Solanum americanum* Miller	*Y’ayit Awut*	1937	Gonorrhoea	Leaves eaten as a vegetable. Root chopped, infused and drunk	E		
Solanaceae							
*Solanum anguivi* Lam.	*Zerch embway*	1938	Scabies		E		Lymphadenitis [4]
Solanaceae			Nosebleed	Root used to brush teeth, the nosebleed will stop	F5		
			Gonorrhoea	Root infusion	E		
*Solanum incanum* L.	*Embway*	2030	Psychiatric disease (*lekeft*) (in formula)	Young shoots (without branch), combined with:	F3		Stomach problem, snake bite, chest pain, tonsillitis, *mich*[68]
Solanaceae			Nosebleed	*Mrenz* root *(Acokanthera schimperi)*			
				*Gumero* root (*Capparis tomentosa*)			
				*Atat* (*Maytenus arbutifolia*)			
				All plants are combined and all the juice is applied through the left nostril. The combination may also be inhaled from smoke	M6	“A nun showed me”	
*Stephania abyssinica* (Quart.-Dill.& A.Rich.) Walp*.*	*Y’ayit joro/Shinet*	2002	Toothbrush	Teeth brushed with the root	M5		Rabies [29]
Menispermaceae							Used in traditional medicine to treat various stomach disorders and syphilis [46]
*Taverniera abyssinica* A. –Rich.	*Dingetegna*		Unexplained stomach ache (*megagna*)	Taken with *Tenadam* (*Ruta chalepensis*) and *Amed* (wood ash), mixed together with a little water and drunk	F1	“Will cause to vomit”	“Sudden disease”, headache, stomach ache [12]
Fabaceae							Vomiting, dysentery [28]
*Thalictrum rhynchocarpum* Dill. Quart.-Dill & A.Rich.	*Serabizu*	2003	Rhesus factor problem in pregnancy (*shotelay*) as part of formula- see *Achyranthes aspera*		M3		Menorrhagia [12]
Ranunculaceae							Urinary tract infection [29]
			As part of hepatitis formula (see *Afrocarpus podocarpus)*		M2		
*Thymus schimperi* Ronniger	*Tosigne*	1955	Whooping cough	Boiled leaves, drunk as a tea	F4	Found on sale in Fiche marketplace	Used medicinally for headaches and coughs [69]
					F5		
Lamiaceae			Hypertension	Boiled leaves, drunk as a tea	F4		
					F5		
*Trigonella foenum-graecum* L.	*Abish*		Demulcent	Mixed with garlic in the treatment of asthma (see *Allium sativum)*, to protect against gastritis which may be caused by strong application of *Allium sativum*	F2	Found on sale in Fiche marketplace	Used in treating skin and stomach disorders [46]
Fabaceae							
*Verbascum sinaiticum* Benth.	*Ye ahiya joro*	2034	Sick chickens	Together with *Feres zeng (Leonotis ocymifolia)*	F1		
Scrophulariaceae			Psychiatric disease (*lekeft*)	In formula (see *Artemisia abyssinica*)	F2		
*Verbena officinalis* L.	*Aqwarach/*	1950	Tonsillitis	Chewed	F5	“My mother would chew it, and she has to take 2 birr* for this. Unless they take the money they cannot be cured. If you refuse, it does not work”	Leaf and/or root juice taken against diarrhoea. Decoction of leaf employed as gargle for tongue disease, sore throat and toothache [70]
	*Attuch/*						
Verbenaceae	*Telenz/*						
	*Hulegeb*						
						*Birr is the unit of currency in Ethiopia	Dysentery, digestive after eating raw meat, eczema, eye disease, heart disease, heart pain, indigestion, induction of diarrhoea and emesis to relieve indigestion, insomnia, liver disease, malaria, mumps, snake/rabid dog bite, sore throat, stomach ache, stomach trouble, tongue disease, tonsillitis [12]
							Stomach disorder, Herpes zoster, ear problems, evil eye, snake bite, ascariasis [28]
*Withania somnifera* (L.) Dunal	*Gizawa*	2033	Unexplained stomach ache (*megagna)*	Root is peeled then used as a fumigant by burning it and inhaling the smoke	M4	“*Gizawa* is my favourite medication. Especially for the stomach. Use the root, peel it, then use it as a fumigant”	Decoction of the root powder taken for rheumatoid arthritis. Bark powder mixed with butter applied as a remedy for swelling [71]
Solanaceae			Bad spirits (*Satan beshita*)	Adaptogen: whole system	F1	“*Gizawa* is an all-out treatment for the whole system”	
			Evil eye (*buda*)		F1	“Gizawa is like salt, it can go with anything. For devil spirit, epilepsy, *buda*. Not for wounds or physical sickness.”	Evil spirit exorcism, joint infection, arthritis, malaria [12]
							Chest pain, *mich,* typhoid, evil eye [68]
					M8, PH	Old saying: “Why did your child die if you had *Gizawa* growing in your garden?”	Narcotic properties. Decoctions are used as pain killers [46]
							Main actions: Adaptogen, antioxidant, antibacterial and antifungal, anti-inflammatory, chondroprotective, anticancer, anxiolytic and antidepressant [72]
*Zehneria scabra* Sond.	*Hareg resa/Shahare*	1954	Dandruff (*forefore*)		F2		Amenorrhoea, intelligence boost, *mich* (fever with headache) [12]
Cucurbitaceae							
			Eye problem (possibly trachoma)	The eyelid is peeled back and rubbed with the back of the leaf. The eyes should be covered and protected from the light until healed.	F1, M8	“The women use it”	*Mich* (fever with headache), stomach ache, wart [49]
					M8		Leprosy, wound dressing, measles, anthelmintic [73]
							*Mich *[28]
							Malaria [29]
*Zingiber officinale* Roscoe.	Zingibil		Taeniasis	As part of formula with *Kosso (see Hagenia abyssinica)*	F1		Widely used as a medicinal plant worldwide
Zingiberaceae			Unexplained stomach ache (*megagna)*	As part of formula with *Ariti* (see *Artemisia absinthium*) and *Tenadam* (see *Ruta chalepensis*)	M3		

Code: M = Male; F = Female; PH = Professional herbalist; E = Ethnobotanist.

Each informant contributed information about the herbs with which they were particularly familiar. Because discussions were allowed to flow in an unstructured way, this did not lead to a fidelity rating for all the herbs as agreement was not specifically sought from each informant on any one herb and no prompts were given. The two occasions where there was significant consensus on use of herbs for specific diseases was in the discussion of herbs for taeniasis and the discussion of the use of *Calpurnia aurea* for childhood diarrhoea (see **Safety**).

### How herbal knowledge was acquired

All of the informants (15) described memories of being treated with herbs for illness as a child. All said they subsequently continued to learn, either from parents or knowledgeable elders, or both (see Table 4)

**Table 4:** How herbal knowledge was acquired

**Informant**	**Exposed to treatment as child**	**Learnt from both parents**	**Learnt only from mother**	**Learnt only from father**	Learnt from others*
Female	6	3		2	1
Male	9	2	2	2	4
**TOTAL**	**15**	**5**	**2**	**4**	**5**

*Others learnt from include “relatives”, “grandmother”, “nun”, “people around the church”.

The two males who had learnt from both parents said that they had learned more from their fathers. One male who learnt only from his mother said that his father had died when he was young. The professional herbalist had learned from both his grandfather (a priest) and his mother.

### Awareness of loss of herbs

There was recognition that some herbs are becoming less accessible, in part due to land degradation and accessibility. When the professional herbalist raised this issue during focus group 2, there was agreement from all present (6 men and 3 women). Examples of comments are:

“*In the old days herbs were everywhere around the house and in the backyard because people planted them, and also they were growing naturally* (referring to the observation in the past that herbs were tolerated or encouraged to grow around human habitation). *Now I have to travel for two days to find some herbs. Even in the forest areas, some don’t exist any more at all*…*Now everyone is looking for herbs, but no-one plants and looks after them*” *(PH)*

“There is degradation of land, deforestation. Marginally the herbs are still available” (F4)

*“Initially the Set eret* (Aloe pulcherrima) *was found close by, but now it is difficult to find this plant, it is only in inaccessible areas now” (M6)*

### Conservation of herbs

Informants demonstrated an understanding of conservation practices in their wildcrafting of the herbs. When *Aloe pulcherrima* plants were dug up during a field-walk/discussion session (W4), the underground stems were planted for future growth, and an informant helping with collection and identification said:

“*We don’t want to take the whole plant because we use that to keep it growing here*” *(M5)*

In a focus group session (FG1), conservative practices were referred to by the professional herbalist*:*

“*Some use six herbs for this [formula]. This means more uprooting of plants. I will use only three herbs for this, that means fewer plants used” (PH)*

### Passing on knowledge

Following a discussion as to whether the younger generation is less likely to be interested in learning about herbal medicine, some informants underscored this issue with their own family experience:

“*Of my 29 children, four (male priests) have been taught. Two of the children of the priests are interested, two are not” (PH)*

“*I have five children. If they are interested, I will pass it on” (M3)*

Community awareness of the threat to the future of traditional herbal medicine has been noted elsewhere in Ethiopia [14].

It has been stated that the younger generation in Ethiopia is increasingly losing interest in learning about the herbs [13,29]. However three children (boys between seven and ten years of age) who joined the field-walk/discussion activities offered some information about the herbs they saw. A nine-year-old boy who worked as a shepherd at the site of a field-walk/discussion excursion, demonstrated in-depth knowledge including recognition and use of medicinal herbs. He was the son of an informant considered a skilled herbalist. The fact that these boys were children of informants, who were knowledgeable about the herbs and used them medicinally, meant that they were more likely to have been exposed to herbal lore in the family setting.

With the possible exception of some herbal medicine education included in religious instruction (there are some known ancient texts held by the Church), due to illiteracy or lack of time, recipes or formulae for herbal treatments continue to be taught to family members solely by demonstration and practical use in the oral tradition of their antecedents.

There is a frequently stated understanding that secrecy is an obstacle to the sharing of knowledge, particularly in the domain of the predominantly male professional herbalists [4,68,74]. In contrast to this, and perhaps reflecting increased awareness of the potential for loss, the professional herbalist at Fiche was keen to be involved and fully supported the Botanica Ethiopia objectives of establishing herbal gardens (Additional file 2), contributing and encouraging discussion and collaboration. When the purpose of the research was explained, he said:

“*Teruneew.* (It is good). *This must happen. What we are doing is important for the herbs”*

Another professional herbalist in the area later supported this statement during a spontaneous conversation. The fact that both herbalists were supportive of the establishment of a community “healing herbs” Association as part of the Botanica Ethiopia initiative, with one of the herbalists becoming Deputy Chairperson of the Association, firmly demonstrated willingness to participate in sharing knowledge.

### Safety

All participants showed awareness of safety issues and dosage importance.

The importance of safety was discussed in relation to dosages of herbs used for contraception, for children, and with herbs known to have strong activity against taeniasis (tapeworm infection). A focus group debate (FG3) centred on the use of the herb *Phytolacca dodecandra* (*Endod*) for contraceptive purposes.

“*I gave this to my wife and she never fell pregnant again. Once you take it you are sterile for life” (M5)*

“*If you spray poison on a flower, it will die*” *(F1)*

Discussions of herbs used for taeniasis showed consensus in the use of certain herbs (FG1, FG2 and FG3), but debate arose around safety in combining the herbs (FG2). Taeniasis is an epidemic infection in Ethiopia, largely due to the custom of eating raw meat [75]. The discussions focused on four herbs: *Glinus lotoides* (*Meterre*), *Embelia schimperi* (*Enkoko*), *Albizia anthelmintica* (*Musena*) and *Hagenia abyssinica* (*Kosso*) with *Guizotia abyssinica* (*Nug*) used as a binder to make a paste with the other herb(s). Informants were concerned about the potential for these herbs to cause toxicity and debated the merits of combining what they described as potent herbs. Each of the informants agreed that the four herbs mentioned were important, but there was disagreement as to whether they should be combined (considered dangerous by some) or used separately, and there were varying opinions on how the herbs should be taken. Table 5 summarises this discussion.

**Table 5:** Discussion of herbs for taeniasis

**Informant** **(M) = Male** **(F) = Female**	**Local names and discussion**	**Botanical names**
F1, F4, F5	*Meterre with Nug*	*Glinus lotoides* + *Guizotia abyssinica* OR
	*OR Musena* with *Nug*	*Albizia anthelmintica* + *Guizotia abyssinica*
	The oil-containing *Nug* seed is ground to a paste and used to mix with the herbs for oral administration	
F2	First preference is *Kechemo*	*Myrsine africana*
	If this does not work, then one of the following	
	a) *Enkoko*. Collect the ripe fruits, remove the outside and swallow the fruit directly using water	*Embelia schimperi*
	OR	
	b) *Musena*, the inflorescence, with *Nug*	*Albizia anthelmintica +Guizotia abyssinica*
	OR	
	c) *Kosso*, the inflorescence with *Nug*	*Hagenia abyssinica + Guizotia abyssinica*
FI	*Kosso* with *Tenadam,* onion, ginger and cinnamon made into *Arake* (spirit brewed with fermented grains)	*Hagenia abyssinica* + *Ruta chalepensis*
M3, M1	*Enkoko* and *Musena*, with *Nug,* combined	*Embelia schimperi + Albizia anthelmintica +Guizotia abyssinica*
M1, M5, M7, M2	*Enkoko*, *Musena* and *Nug* – to be taken with Tella (a traditional drink made from grains), or there will be a reaction	*Embelia schimperi + Albizia anthelmintica + Guizotia abyssinica*
M1, M7, M8	“Taking *Musena* and *Enkoko* together can be dangerous”	*Albizia anthelmintica + Embelia schimperi*

In this context, it is interesting to look at whether there has been exploration of the use of these herbs for taeniasis elsewhere. Animal and *in vitro* studies have been conducted on *Glinus lotoides, Embelia schimperi, Albizia anthelmintica and Hagenia abyssinica.* In 2006 a paper demonstrating the safety of *Glinus lotoides* as a taenicidal herb was published [76] but a subsequent investigation showed potential for genotoxicity in mice [77]. There have been investigations into the toxicity and therapeutic activity of a number of herbs traditionally used for taeniasis, including the herbs mentioned by the group in Fiche: *Albizia anthelmintica*, *Embelia schimperi, Glinus lotoides, Hagenia abyssinica* and *Myrsine africana*[75,78-80]. One of these studies reported *Myrsine africana* to have ‘lethal action against tapeworm’ [79]. The repeated mention by the informants of this group of herbs in the context of treatment of tapeworm infection contributes to existing documentation of their traditional usage in Ethiopia [3,17,30,75,77] and warrants further pharmacological investigation for their medicinal value.

Another example of a discussion of herbal safety occurred in a focus group (FG3) and concerned the use of *Calpurnia aurea* (*Digita)* for the treatment of childhood diarrhoea. The dosage, strength and potential toxicity of this herb were discussed.

“*Take the young shoots from seven plants of* Digita*, rub the leaves in the hands for juice, for children with diarrhoea (*tekmat*). Put juice into water depending on the age of he child, dosage is very important. It is very strong. Very small by spoon. One teaspoon. Just once” (F3)*

“*It can be very dangerous. They* [informants] *say the stem bark is poisonous. Only the young shoots are used and even then one has to be very careful” (M8, also translating)*

“*Actually it can send you crazy. If you go crazy, you will die” (F1)*

“It should be measured carefully” (F2)

The use of *Calpurnia aurea*, a quinolizidine alkaloid-containing member of the family Fabaceae, for the treatment of diarrhoea and a range of other conditions, is well documented from Ethiopia and other parts of Africa [28,45]. It has demonstrated anti-diarrhoeal effect in mice and *in vitro* inhibitory activity against a range of diarrhoea-causing bacteria [45].

### Gender

The literature frequently discusses the Ethiopian tradition of preferentially passing on knowledge in the male line, either through the Church tradition or within the family [3,12,17,81] and studies tend to show that men have better medicinal plant knowledge [4]. However in focus group 1 (5 men and 1 woman), when one of the men declared that women hold more knowledge, all agreed that women have more herbal knowledge than men relating to the use of medicinal plants in the home.

“*In the countryside, the women hold all the knowledge…the women had to learn the hard way, because men could be away at war or simply not there, so the women left behind have to take care of themselves and their children” (M1, with agreement from PH, M3, M4, M8, F1)*

“More women know about the application of the herbs” (PH)

This concurs with the findings of Fassil in her 2005 study of home-based medicinal plant use in rural communities in the Bahir Dar Zurie Wereda (district) in northwest Ethiopia [9], which showed that women have particular roles in traditional health care delivery in their capacities as mothers and cultivators of home gardens, and also the 2003 study by Gedif and Hahn [17] which recruited mothers as informants.

Group discussions were not so effective at capturing the information of the women as they were often pressed for time and unable to be present for as long as the men. Even during interviews the women were busy with children or food preparation. This limitation was also noted by Fassil [9].

### Herbs: identification and usage

Both men and women on the field-walk/discussion activities demonstrated ability to identify medicinal herbs. At the Saturday market, women were the vendors of the herbs and were knowledgeable about their uses. The Saturday market was attended by members of the Fiche community and surrounding towns, with a variety of stalls managed by men, women and children selling foodstuffs (including culinary herbs), household equipment, and medicinal herbs. From a survey taken at the Saturday market, 15 medicinal herbs were identified (Table 6).

**Table 6:** Market Survey Herbs identified and information collected from vendors of medicinal plants

**Botanical name**	**Amharic name**	**Use**	**Comment**
*Artemisia absinthium*	*Ariti*	Stomach ache	Observed in a fragrant bouquet with Tej sar *(Cymbopogon citratus)* and Ajuban *(Ocimum basilicum var. thyrsiflorum)*
*Echinops kebericho*	*Kebericho*	Used as incense in the home for children who have nightmares	Smoke may be inhaled by covering head with blanket over the smoking root
		Prevents nocturia	
		Repels snakes from house	
*Glinus lotoides*	*Meterre*	Taenicidal	Cleaned, ground with Nug (*Guizotia abyssinica)*. Taken with a little sugar and eaten before food. Necessary to fast until noon prior to administering
			1 birr for a small cup
*Guizotia abyssinica*	*Nug*	Ground seed used as binder	Seed is ground to a paste and mixed with herbs for administering
*Lepidum sativum*	*Feto*	Abdominal pain	Chopped, infused and drunk
*Lippia adoensis* var. koseret	*Koseret*	Culinary – used in making *niter kibbe* (a type of ghee)	
*Nigella sativa*	*Tikurazmud*	Culinary	
*Ocimum basilicum* var. *thyrsiflorum*	*Ajuban*	Fragrant bouquet	
		Culinary flavouring/spice	
*Olea europea*	*Weyra*	Sterilising treatment for milk equipment	Oil collected from root
*Otostegia fruticosa*	*Tenjut*	For stomach pain	The leaves are burnt and smoke inhaled
		The branch is used for cleaning teeth	
*Rhamnus prioides*	*Gesho*	To make *Tella* (fermented drink)	
*Rosmarinus officinalis*	*Asmarino, Yetibs ketel*	Culinary flavouring, hair rinse	
*Ruta chalepensis*	*Tenadam*	Megagna (stomach pain)	Used in coffee
*Thymus schimperi*	*Tosign*	Hypertension	
*Trigonella foenum-graecum*	*Abish*	Digestive and culinary, skin disorders	

## Conclusion

This study has shown that herbal medicine continues to be of great importance to this community in Ethiopia as part of their healthcare system, and they are aware that the knowledge and the herbs are at risk of disappearing. Knowledge continues to be passed on via the oral tradition and by application. This community is motivated to help to increase awareness of, and accessibility to, the herbs they use to treat illness in the family home.

There were several important aspects noted during this study that future researchers in the area may wish to consider. One recommendation arising from our experience is that women be released from domestic duties for the purpose of interviews and focus groups. This would allow them to contribute their knowledge and experience more fully.

It may fairly be argued that conducting a study where the principal researcher does not share language or cultural background could present significant obstacles, but there were unexpected advantages that arose from this. The researcher’s presence demonstrated to the informants an external awareness of, and respect for, the knowledge held by the community, and for their predicament. The fact that the research supported the implementation of a project to establish a medicinal herb garden in the community also contributed to the willingness of the informants to contribute and share their knowledge. Collaboration with Ethiopian authorities (AAU and EIB) was essential for the successful conduct of the research. It was also important and helpful to consult with local authorities. Local government (*Kebelle*) and City Council representatives provided administrative support for the formation of the Etse-Fewus (Healing Plants) Association subsequent to the fieldwork, and local government subsequently donated land for a community medicinal garden, giving demonstrable government legitimacy to the initiative.

We recognise that all these elements were critically important for the successful conduct of the research and future researchers are encouraged to investigate how they may best support the communities with which they work. In doing so, they will contribute in part to the United Nations Millennium Development Goals [36], primarily those related to reduction of child mortality, improvement of maternal health, combating HIV/AIDS, malaria and other diseases, promoting gender equality and empowering women, and ensuring environmental sustainability.

If Ethiopians lose their traditional herbal medicine - either the knowledge, or the plants or both - they will lose the ability to provide herbal treatment for their families. If they are also unable to access conventional medicine either through lack of affordability or availability, as is still the case in many rural areas particularly, they would be in an unenviable situation. Ethnobotanical, ethnomedical and anthropological research must continue in Ethiopia in order to understand the cultural, sociological and practical considerations that inform the wider community at institutional and governmental level. In the future, Ethiopians should be able to take advantage of opportunities to develop the potential of their rich medicinal plant resources via documentation of knowledge of use and pharmacological investigation of medicinal properties of the plants. Integration of traditional herbal medicine with outreach medical services may be a beneficial outcome of supporting further investigations in Ethiopia’s medicinal herb lore.

## Abbreviations

AAU: Addis Ababa University; EIB: Ethiopian Institute of Biodiversity; SCU: Southern Cross University.

## Competing interests

The authors declare that they have no competing interests.

## Authors’ contributions

Ed’A conceived of the study, carried out the fieldwork and drafted the manuscript. HW supervised the research and contributed to the manuscript. ZA helped with internal in-country support and guidance, providing links within AAU and with EIB and arranging collaboration with AAU, and discussing the scientific issues, giving guidance throughout. TA assisted with translation during data collection, and collected and prepared voucher specimens that were lodged at EIB herbarium. All authors read, enriched and approved the final manuscript.

## Authors’ information

Elizabeth d’Avigdor, DMH, Dip. Nutr. M.Cl.Sc. (Comp. Med). Herbalist and nutritionist, NSW Australia. Ms d’Avigdor conducted the research in Ethiopia and Australia as part of her postgraduate studies at Southern Cross University, and developed the “Botanica Ethiopia: A Living Pharmacy” project in joint partnership with Global Development Group. Email edavigdor@hotmail.com, http://www.botanicaethiopia.com.

Dr. Hans Wohlmuth, a) Division of Research, Southern Cross University, Lismore, NSW 2480, Australia, and b) Integria Healthcare, Gallans Road, Ballina, NSW 2478, Australia.

Dr. Zemede Asfaw, Associate Professor of Ethnobotany, Department of Plant Biology & Biodiversity Management, College of Natural Sciences, Addis Ababa University, P.O. Box 3434, Addis Ababa, Ethiopia.

Dr. Tesfaye Awas, Botanist, Ethiopian Institute of Biodiversity, PO Box 30726, Addis Ababa, Ethiopia.

## Supplementary Material

Additional file 1 Film footage. Click here for file

Additional file 2 Botanica Ethiopia project description. Click here for file
