# Supplementary material for: The current status of knowledge of herbal medicine and medicinal plants in Fiche, Ethiopia
Source: J Ethnobiol Ethnomed. 2014 May 6;10:38. doi: 10.1186/1746-4269-10-38 (PMC4068905; doi:10.1186/1746-4269-10-38)
Supplement: Additional file 2 — Botanica Ethiopia project description. [file 1746-4269-10-38-S2.docx]

**Additional File B**

**J655N Botanica Ethiopia: A Living Pharmacy**

**Project Description**

The J655N Botanica Ethiopia: Living Pharmacy project is delivered in partnership with Global Development Group, an Australian Non-Government Organisation that carries out humanitarian projects and provides long term solutions to poverty. The project was developed by Elizabeth d'Avigdor with support from Blackmores Ltd. Australia, and following her postgraduate research in Ethiopia which was conducted with the assistance of Southern Cross University Australia, Addis Ababa University, Ethiopia, and the Ethiopian Institute of Biodiversity, Ethiopia. Working with the Fiche community and local partners the ‘Living Pharmacy’ project focuses on how traditional herbal medicine can continue to support the health of Ethiopian families and the wider community.

**PURPOSE**

The purpose of this project is to support the practice of traditional herbal medicine in Fiche, Ethiopia through the establishment of a sustainable indigenous herbal medicine garden within the Fiche community of Ethiopia.

**PROJECT BACKGROUND**

**The Global Perspective on Traditional Medicine**

The World Health Organisation (WHO) defines traditional medicine as “the sum total of knowledge, skills and practices based on the theories, beliefs and experiences indigenous to different cultures that are used to maintain health, as well as to prevent, diagnose, improve or treat physical and mental illness”[[1](#_ENREF_1)]. The WHO calls for the inclusion of traditional medicine in primary health care services, emphasizing the benefits to wider numbers of people, particularly in rural areas, of the availability and low cost of medicinal plants.

At the 2008 WHO Congress on Traditional Medicine, 70 member states adopted the Beijing Declaration, a statement of intention to promote the safe and effective use of traditional medicine, and to take steps to integrate traditional medicine into national health systems[[2](#_ENREF_2)] .

Globally, there is strong support for the encouragement of traditional medicine practices for a number of reasons:

- The imperative to preserve indigenous knowledge of traditional medicine practices which are currently endangered due to the lack of written documentation of such practices, the lack of further education and lack of resources.
- The need to support local economies in countries where traditional medicine is practiced and used as a primary form of health care.
- The global concern to protect plant species in danger of extinction and protect natural resources.

In regards to Africa, the support of traditional medicine practices is best summarised by the WHO African Region Mission Statement on the WHO website: “The Regional Programme on Traditional Medicine has the responsibility to collaborate with Member States to promote the rational use of traditional medicine within sustainable health care delivery systems, bearing in mind specific socio-cultural environments”[[3](#_ENREF_3)].

**United Nations Millennium Development Goals and Ethiopia**

The eight Millennium Development Goals (MDGs) were developed from the eight chapters of the United Nations Millennium Declaration, under the aegis of the United Nations Development programme, and were signed by 189 countries, in September 2000. Each country tailored the MDGS to suit its own specific development needs. The Goals provide a framework for the international community to work together to achieve a significant reduction in world poverty.

The goals are:

- Eradicate extreme poverty and hunger
- Achieve universal primary education
- Promote gender equality and empower women
- Reduce child mortality
- Improve maternal health
- Combat HIV/AIDS, Malaria and other diseases
- Ensure environmental sustainability
- Global partnership for development

[[4](#_ENREF_4)]

At the United Nations Economic and Social Council Annual Ministerial Review of 2007, the Government of Ethiopia presented its Poverty Reduction Strategies (PRS) as a primary vehicle for achieving the Millennium Development Goals. The Plan for Accelerated and Sustained Development to End Poverty (PASDEP), one of the PRS, stated the following aims:

- Building all-inclusive implementation capacity
- A massive push to accelerate growth
- Creating the balance between economic development and population growth
- Unleashing the potentials of Ethiopia's women
- Strengthening the infrastructure backbone of the country
- Strengthening human resource development
- Managing risk and volatility
- Creating employment opportunities.

[[5](#_ENREF_5)]

This project supports aims of the Millennium Development Goals and the Ethiopian Poverty Reduction strategies by:

- Supporting sustainable herbal medicine practices
- Protecting species of flora that may be endangered and promoting environmental education regarding local flora (medicinal herbs)
- Enabling and encouraging women to participate
- Providing access to traditional medicine health care

**Traditional Medicine Practices in Ethiopia**

With 80% of the population of Ethiopia depending on traditional medicine practices for primary health care [[2](#_ENREF_2)], understanding the historical and cultural aspects of traditional medicine use in Ethiopia is essential for the successful implementation of modern practices. Efforts are being made by the government of Ethiopia to institute policies to address the issue of loss of traditional knowledge and to further research the pharmaceutical usefulness of indigenous herbs. For example:

- The Ethiopian Ministry of Health Website states that one of its mandates is: “to create conducive conditions for research, registration and utilisation of traditional medicines and give the necessary support to practitioners to register and practice in their field” [[6](#_ENREF_6)]

**The Medicinal Herb Garden**

The aim of a community medicinal herb garden is to provide knowledge about, and access to, the indigenous medicinal herbs used to treat common complaints in the domestic sphere. There are three significant reasons why this is important:

- *Preservation of knowledge:* The knowledge of traditional herbs and traditional herbal medicine practice is in danger of extinction because it has been little documented, owing largely to the oral tradition of passing on knowledge and the level of secrecy surrounding traditional medicine practices. Even the oral tradition has been losing ground because the expansion of modern education is having an impact on traditional family ties and traditions, including the passing on of traditional knowledge.
- *Preservation of herb species:* Another important benefit of the community medicinal garden is to preserve herb species in danger of extinction from wildcrafting. There is some minimal effort by local THP to cultivate home gardens, but without support and resources (such as land and funds) it is apparent the practice will not achieve a wider consistent benefit of conservation of species.
- *Access to health care:* 80% of the population of Ethiopia use traditional medicine as a primary form of health care. This means that traditional medicine practices and herbal medicine are extremely important to the health of most people in rural areas particularly (where there may be no access to a doctor), providing a first response to a health problem at a household level [[7-9](#_ENREF_7)]

**Successful Programmes**

The “Home Herbal Garden” (HHG) community projects in India, Africa and Brazil are projects that have evolved as a means to:

- Alleviate poverty by providing employment and income opportunities
- Educate a community regarding their indigenous medicinal herbs
- Preserve traditional medicine practices
- Provide health care that may not otherwise be available
- Protect species of flora that may be endangered
- Provide opportunities for women

**India**

India has a similar background to Ethiopia in terms of use of traditional health care practices. 65% of the total population does not have access to modern health care, using local medicinal plant knowledge systems as their primary health care. At the community level, the programmes related to medicinal plant and associated local knowledge systems are organized by local NGOs and community groups led by women. One of these is the Foundation for Revitalisation of Local Health Traditions (FRLHT).

Based in Bangalore, the efforts of the FRLHT’s prize-winning Home Herbal Garden programme has resulted in the implementation of 190,000 home gardens throughout India. Women across more than 9500 villages have been trained to instruct the neighbouring households on self-help methods of preparing medicinal plant-based home remedies to relieve common ailments. The gardens are used to meet the primary health care needs of some of the poorest households in India.

The HHG programme presented a cost-effective strategy to empower the rural communities in their health practices; in addition, health and economic benefits are shared by friends and neighbours [[10](#_ENREF_10)]

FRLHT has been recognised by the Indian Government as a ‘centre of excellence’ in the field of medicinal plants.

**Brazil**

Plantas Do Nordeste, in collaboration with the Royal Botanic Gardens at Kew, UK, has developed a dissemination and training project on economically useful plants for rural communities in Northeastern Brazil. The aim of this programme is to provide affordable medicines, of proven pharmacological efficacy, for low-income socioeconomic groups. This was achieved through the cultivation of scientifically validated medicinal plants selected from those traditionally used in the Northeast of Brazil.

Approximately 16 ‘Living Pharmacies’ were established throughout Ceara and other states. These medicinal plant gardens provided a learning tool for members of local communities who were taught how to tend the plants and produce teas, lotions and pills. Leaflets containing this information were produced and distributed to local communities at workshops and clinics.[[11](#_ENREF_11)]

**Africa**

**Ethiopia**

The Ethiopian Institute of Biodiversity Conservation was set up to promote the development and sustainable utilisation of the country’s biological resources. A stated objective of the Institute is to conserve and promote sustainable utilisation of medical plant genetic resources and associated indigenous knowledge for prevention and treatment of human and animal diseases.

**South Africa**

Garden Africa is a UK-based charity whose work is focused in Southern Africa and whose aim is to establish productive organic training gardens in schools, hospitals and clinics, growing nutritious foods and medicinal herbs.

The Africulture Project, located on the outskirts of Grahamstown, is one of Garden Africa’s initiatives and is aimed at enabling poor and marginalised people to meet their basic needs by promoting the sustainable use of medicinal plants and traditional healthcare knowledge. One in three people in Grahamstown are HIV positive, and local medicinal plants are recognised for their effectiveness as immune and appetite stimulants.

In partnership with the Royal Botanic Gardens, Kew, and with strong institutional support from within South Africa, the one-hectare training facility and nursery specializes in the cultivation, processing and marketing of traditional plants. An important aspect of the project is the dissemination of information on drug interactions between pharmaceutical medicines and medicinal plants amongst traditional healthcare practitioners. [[12](#_ENREF_12)]

**Project goals**

1. To create a sustainable and replicable indigenous herbal medicine garden by employing best practice models
2. To build the capacity of the Fiche community
3. To provide a replicable model for other communities

**Project objectives per goal**

*GOAL 1: To create a sustainable garden by employing best practice models*

1. Use current best practice models through research, for building a sustainable herbal medicinal garden
2. Build a garden in the Fiche community using Ethiopian herbs traditionally used for domestic use in treating common ailments

*GOAL 2 : To build the capacity of the Fiche community*

1. Share knowledge with and support dissemination of knowledge amongst, the Fiche community about the benefits of medicinal herbs
2. Provide the potential for stakeholders to earn income from the sale of medicinal herbs at local markets

*GOAL 3 : To provide a replicable model for other communities*

1. Provide a how-to manual as a template for other communities
2. Open display garden for visits by other community representatives

**Project outcomes**

1. A sustainable herbal medicine garden for use by the Fiche community in treating basic health conditions.
2. A “How To” manual for the establishment of a sustainable herbal medicine garden translated in Amharic and English.
3. A replicable project model that can be taken up in neighbouring communities within Ethiopia
4. Potential income building for the Fiche community from the sale of herbs and herb products at local markets

**Achievements to date**

Research conducted in Ethiopia performed two functions: i) it identified the need for preserving knowledge and herbs at risk of loss, and ii) it identified the willingness and capacity of the local community to take steps towards preservation.

With the encouragement and support of Botanica Ethiopia, the Fiche community who were involved in the research have formed the “Etse-Fewus (Healing Herbs) Association, with the support of Local Government and City Council. This Association committed to developing their home household herbal gardens, and as a direct result the Local Government and City Council donated land for a large community garden. This land is now fenced, the soil developed and planting has commenced of herbs which were identified in the research as being of local use and benefit, some of which are becoming less accessible due to land degradation, increasing industry and other environmental issues.

**Vision for the future**

The scope of this project is intended to go further in the future: there are possibilities once the garden is well established for international visits from ethnobotanical groups, permaculture groups and herbal medicine/complementary medicine practitioners. The Etse-fewusAssociation is investigating the possibility of developing bee-keeping activities In this way the sustainability of the project is further established and will bring interest and further capacity building for the community. It is envisaged that the success of this project will inspire and support replication of the initiative in other areas of Ethiopia and elsewhere in the world.

The J655N Botanica Ethiopia: A Living Pharmacy project start-up was funded by Blackmores Ltd. Further funds were provided by AACASA (Australian African Childrens’ Aid Support Organisation) and individual donations .

A website has been developed by journalist May Slater to document the progress of the project: [www.botanicaethiopia.com](http://www.botanicaethiopia.com)

**1.** Traditional Medicine: Fact Sheet 134 **[**<http://www.who.int/medicines/areas/traditional/en/>**]**

**2. WHO: WHO Congress on Traditional Medicine and the Beijing Declaration.** *WHO Drug Information* 2009, **23:**8-11.

**3.** Traditional Medicine Programme: Mission and Functions **[**<http://www.afro.who.int/trm/index.html>**]**

**4.** United Nations Development Programme. Millennium Development Goals **[**<http://www.undp.org/mdg/basics.shtml>**]**

**5.** United Nations Economic and Social Council: Plan for Accelerated and Sustained Development to End Poverty (PASDEP). United Nations Economic and Social Council; 2007.

6. Ethiopian Ministry of Health: Mandates of the Federal Ministry of Health. 2009.

**7.** Gedif T, Hahn H: **Treatment of Malaria in Ethiopian folk medicine.** *Trop Doct* 2002**,** 32:206-209.

**8.** Abebe D, Hagos E: **Plant Genetic Resources of Ethiopia.** In *Plants as a primary source of drugs in the traditional health practices of Ethiopia.* Edited by Engels J, Hawkes J, Worede M. Cambridge: Cambridge University Press; 1991: 101-113

**9.** Teklehaymanot T: **Ethnobotanical study of knowledge and medicinal plants use by the people in Dek Island in Ethiopia.** *J Ethnopharmacol* 2009, **124**:69-78.

**10.** Bodeker G, Burford B: *Traditional, complementary and alternative medicine: policy and public health perspectives.* Imperial College Press, London; 2007.

**11.** The "Top 10" Project - a response to the needs of local communities [<http://www.kew.org/science/pne/pne14_engl.pdf> Accessed August 2009]

**12.** Garden Africa [<http://www.gardenafrica.org.uk/about_us.htm>]
